# Advances of Community-Level Plant DNA Barcoding in China

**DOI:** 10.3389/fpls.2017.00225

**Published:** 2017-02-21

**Authors:** Nancai Pei, Bufeng Chen, W. J. Kress

**Affiliations:** ^1^Key Laboratory of State Forestry Administration on Tropical Forestry Research, Research Institute of Tropical Forestry, Chinese Academy of ForestryGuangzhou, China; ^2^Department of Botany, MRC-166, National Museum of Natural History, Smithsonian InstitutionWashington, DC, USA

**Keywords:** DNA barcode, community phylogeny, reproductive trait, forest biology, subtropical forest

## Abstract

DNA barcoding is a commonly used bio-technology in multiple disciplines including biology, environmental science, forensics and inspection, etc. Forest dynamic plots provide a unique opportunity to carry out large-scale, comparative, and multidisciplinary research for plant DNA barcoding. The paper concisely reviewed four previous progresses in China; specifically, species discrimination, community phylogenetic reconstruction, phylogenetic community structure exploration, and biodiversity index evaluation. Further, we demonstrated three major challenges; specifically, building the impetus to generate DNA barcodes using multiple plant DNA markers for all woody species at forest community levels, analyzing massive DNA barcoding sequence data, and promoting theoretical innovation. Lastly, we raised five possible directions; specifically, proposing a “purpose-driven barcode” fit for multi-level applications, developing new integrative sequencing strategies, pushing DNA barcoding beyond terrestrial ecosystem, constructing national-level DNA barcode sequence libraries for special plant groups, and establishing intelligent identification systems or online server platforms. These efforts will be potentially valuable to explore large-scale biodiversity patterns, the origin and evolution of life, and will also facilitate preservation and utilization of biodiversity resources.

## Introduction

DNA barcoding, a bio-technology characterized by standardization, universality and efficiency (Hebert et al., 2003), is widely used in multiple disciplines including biology, environmental science, forensics and cross-boarder inspection, etc. DNA barcoding technology may be most promising to achieve the goals of rapid and accurate species identification and sustainable utilization of biological resources (CBOL Plant Working Group, 2009; Janzen et al., 2009; Harris and Bellino, 2013; Hollingsworth et al., 2016). Meanwhile, many drawbacks and problems cannot be neglected with use of DNA barcodes, including failure of amplification or sequencing, difficulties in finding universal primers, lack of barcoding gap, hybridization and introgression in some plant groups (Collins and Cruickshank, 2013; Zinger and Philippe, 2016). Concerns of plant DNA barcoding may differ in biologists; specifically, taxonomists mainly focus on clades, systematists on phylogenies, while ecologists on communities.

China initiated its national-level project of DNA barcoding (including animals, plants, and microbes) in 2008, and was invited as one of the four iBOL (International Barcode of Life^1^) central node nations in the world (Che et al., 2010). Vegetation types in China are extremely diverse involving tropics, subtropics and temperate zones. Forest dynamic plots (FDPs), according to the standard Smithsonian CTFS/ForestGEO protocol (Condit, 1998) and also conducted based on Forestry Standards for “Observation Methodology for Long-term Forest Ecosystem Research” of the People’s Republic of China (LY/T 1952-2011), provide us an opportunity to study large-scale and multidisciplinary research on forests including DNA barcoding. Presently, 15 FDPs were set up across the Chinese mainland (13 belonging to CForBio monitoring network^2^; plus Heishiding FDP in Guangdong province, and Jianfengling FDP in Hainan province). Additionally, Tai-Po-Kau FDP in Hong Kong and three FDPs (i.e., Lienhuachih, Fushan, and Nanjenshan) in Taiwan were also set up successively. Nowadays, there are four, nine, and six FDPs in tropics, subtropics and temperate zone, respectively. At least seven FDPs (i.e., Dinghushan, Gutianshan, Changbaishan, Xishuangbanna, and three FDPs in Taiwan) are available with plant DNA barcoding sequences (mostly *rbc*L, *mat*K, *trn*H-*psb*A, and occasionally ITS2). Additional two FDPs are underway to release or publish DNA barcoding sequence data (e.g., Badagongshan in Hunan province, Jianfengling in Hainan Island, etc.).

Based on Dinghushan FDP (a lower subtropical forest in south China), a Chinese research team cooperating with international scientists, initiated a plant DNA barcoding project (i.e., community-level) in 2008. The first research article was published in 2011, utilizing a well-resolved DNA barcode phylogeny to explore tree-habitat associations. Subsequently, approximately a dozen international and domestic publications of closely related or comparative topics were available in China (Pei et al., 2014). Besides of contributions from Chinese forests, studies on plant DNA barcoding involving forest communities are published worldwide across tropics, subtropics and temperate zones (Gonzalez et al., 2009; Kress et al., 2009, 2010; Burgess et al., 2011; Parmentier et al., 2013; Saarela et al., 2013; de Boer et al., 2014). Generally, applications of plant DNA barcoding can be divided into clade and community levels, and this paper focuses on the latter. We developed selection criteria that all investigations should be dominated by or participated with Chinese researcher(s) and included publications be carried out in one or more Chinese forest communities. Here, we briefly demonstrated recent progress, major challenges, and possible future directions of the community-level plant DNA barcoding in China.

## Previous Advances

(1) Species discrimination had become easy and rapid by means of DNA barcoding technology. Generally, the single DNA barcoding marker *trn*H-*psb*A raised relatively high rates of species discrimination, followed by *mat*K and *rbc*L. The combination of *rbc*L+*mat*K (a core barcode for land plants recommended by CBOL Plant Working Group) averagely discriminated 88.6, 83.8, and 72.5% at the local, regional and global scales, respectively (Pei et al., 2015a). An additional intergenic spacer, either *trn*H-*psb*A or ITS2, had also proven to be useful in different taxonomic groups (China Plant BOL Group et al., 2011; Liu et al., 2015; Pei et al., 2015a). Rates of species discrimination varied along a latitudinal gradient and were negatively correlated with ratios of closely related taxa and generally depended on geographic scales in global FDPs (Pei et al., 2015a).

(2) Community phylogenetic reconstruction, via a super-matrix approach with the three-locus barcode combination (*rbc*L+*mat*K*+psb*A-*trn*H) provided a well-resolved phylogenetic framework. Thus, a DNA barcoding-based phylogeny could assign almost all species to a proper evolutionary position in a systematic classification (Pei, 2012; Erickson et al., 2014), when compared to “Phylomatic phylogenies,” which were usually accompanied by more polytomies and resulted in a larger bias of phylogenetic community structure. In addition, a powerful R-package named “phylotools” (Zhang et al., 2012) had been developed to build a super-matrix for multi-locus DNA barcodes, and easily calculated the inequality among lineages and phylogenetic similarity for large datasets.

(3) The exploration of phylogenetic community structure benefited greatly from well-resolved phylogenies generated from plant DNA barcodes. Phylogenetic signal has been detected in plant-habitat associations (i.e., closely related species tend to prefer similar habitats; Pei et al., 2011). In addition, patterns of co-occurrence within habitats and functional traits across spatial and size scales were typically non-random with respect to community phylogenies and gave strong support for a deterministic model rather than for a neutral model (Pei et al., 2011; Yang et al., 2014).

(4) Biodiversity index evaluation proved to be more effective and comparable with the aid of standardized plant DNA barcoding procedures. Phylobetadiversity might not be significantly affected by species abundance when scales were relatively small, which might result from reductions in evenness in communities as scales increased. Moreover, *phylogenetic* measures of alpha and beta diversity were not strong predictors of *functional* alpha and beta diversity, but partitioning the variation in phylogenetic and functional beta diversity showed that environmental distance was generally a better predictor of beta diversity in diverse forests worldwide (Feng et al., 2012; Swenson et al., 2012; Yang et al., 2015). Overall, in view of cost-effectiveness and the trade-off between sequence recovery and species resolution, we suggested the combination of markers *rbc*L+*mat*K+*trn*H-*psb*A as a priority for DNA-based studies on forest communities (Pei et al., 2015a).

## Major Challenges

Though numerous important progress had been made in the past 10 years, we raised that the following three challenges might be tough but remain promising: (1) Building the impetus to generate DNA barcodes using multiple plant DNA markers for all woody species at forest community levels, which requires significant research resources and investment; (2) Analyzing massive DNA barcoding sequence data, which needs powerful computational systems and critical infrastructures to perform large-scale and multidisciplinary research projects; and (3) Promoting theoretical innovation, which calls for raising novel scientific hypotheses and publishing a series of influential papers in top academic journals.

## Future Directions

Comparative analyses of community phylogenies from forest dynamics plots or natural reserves are feasible owing to the universal and standard working routine of plant DNA barcoding (Kress and Erickson, 2012). When combined with conservative plant traits (e.g., flowering phenology; Pei et al., 2015b), effects of individuals on assembly patterns within communities, dissimilarity of diverse communities along an environmental gradient, and non-random processes therein should be more thoroughly explored. Possible directions are: (1) Proposing a “purpose-driven barcode” (e.g., metabarcoding and mini-barcode) fit for multi-level applications such as identifying living organisms, reconstructing community phylogenies, detecting environmental biodiversity information, and exploring ecological network structure (Little, 2014; Kress et al., 2015; Evans et al., 2016); (2) Developing new integrative sequencing strategies (e.g., genome skimming; Hollingsworth et al., 2016) to generate mega-phylogenies in face of the post-genomic era; (3) Pushing DNA barcoding beyond terrestrial ecosystem, to include aquatic ecosystem (including mangroves; Richards and Friess, 2016) with complicated biotic interactions and abiotic extreme conditions to explore mechanisms of formation, maintenance, and evolution; (4) Constructing national-level DNA barcode sequence libraries of economically valuable tree species for commercial authentication and endangered plant taxa against illegal international trade (Xu et al., 2015; Zhao et al., 2015); and (5) Establishing intelligent identification systems (e.g., Leafsnap) or online server platforms (e.g., i-Flora and e-Flora; Kumar et al., 2012; Li et al., 2012; Zeng et al., 2014; Pei, 2016) for land plants integrating genetic, morphological and environmental information, which will make DNA-based plant identification more precise, more convenient, and more interesting. These pursuits will be valuable to explore large-scale biodiversity patterns, the origin and evolution of life, and will also facilitate preservation and utilization of biological resources. We hope that this paper will be an addition to the field.

## Author Contributions

NP conceived and wrote the draft, NP, BC, and WJK revised the paper and approved it for publication.

## Conflict of Interest Statement

The authors declare that the research was conducted in the absence of any commercial or financial relationships that could be construed as a potential conflict of interest.

## Footnotes

1 http://ibol.org/

2 http://www.cfbiodiv.org/
